# Comparative phosphoproteomics reveals evolutionary and functional conservation of phosphorylation across eukaryotes

**DOI:** 10.1186/gb-2008-9-10-r144

**Published:** 2008-10-01

**Authors:** Jos Boekhorst, Bas van Breukelen, Albert JR Heck, Berend Snel

**Affiliations:** 1 Bioinformatics, Department of Biology, Faculty of Science, Utrecht University, Padualaan, 3584 CH, The Netherlands; 2 Biomolecular Mass Spectrometry and Proteomics Group, Bijvoet Center for Biomolecular Research and Utrecht Institute for Pharmaceutical Sciences, Utrecht University, Sorbonnelaan, 3584 CA Utrecht, The Netherlands; 3 Academic Biomedical Centre, Utrecht University, Yalelaan, 3584 CL Utrecht, The Netherlands

## Abstract

**Background:**

Reversible phosphorylation of proteins is involved in a wide range of processes, ranging from signaling cascades to regulation of protein complex assembly. Little is known about the structure and evolution of phosphorylation networks. Recent high-throughput phosphoproteomics studies have resulted in the rapid accumulation of phosphopeptide datasets for many model organisms. Here, we exploit these novel data for the comparative analysis of phosphorylation events between different species of eukaryotes.

**Results:**

Comparison of phosphoproteomics datasets of six eukaryotes yields an overlap ranging from approximately 700 sites for human and mouse (two large datasets of closely related species) to a single site for fish and yeast (distantly related as well as two of the smallest datasets). Some conserved events appear surprisingly old; those shared by plant and animals suggest conservation over the time scale of a billion years. In spite of the hypothesized incomprehensive nature of phosphoproteomics datasets and differences in experimental procedures, we show that the overlap between phosphoproteomes is greater than expected by chance and indicates increased functional relevance. Despite the dynamic nature of the evolution of phosphorylation, the relative overlap between the different datasets is identical to the phylogeny of the species studied.

**Conclusion:**

This analysis provides a framework for the generation of biological insights by comparative analysis of high-throughput phosphoproteomics datasets. We expect the rapidly growing body of data from high-throughput mass spectrometry analysis to make comparative phosphoproteomics a powerful tool for elucidating the evolutionary and functional dynamics of reversible phosphorylation.

## Abstract 2

A comparison of phosphoproteomics datasets of six eukaryotes shows significant overlap between phosphoproteomes.

## Background

Post-translational modifications play important roles in a wide range of cellar functions. Reversible phosphorylation has been studied extensively and is known to influence protein function by changing protein-protein binding properties, activity, stability, and spatial organization [1]. Phosphorylation plays a key role in signal transduction cascades [2] and allows the fine tuning of protein complex assembly [3]. It is estimated that about one-third of all proteins in eukaryotic cells are phosphorylated at any given time [1].

Recent developments in high-throughput phosphoproteomics studies have resulted in the availability of phosphopeptide datasets for many model organisms. As a result, tools for the comparison of phosphoproteomes are emerging [4]. Although these high-throughput datasets do not capture all phosphorylated peptides of a species under a given condition, large advances in enrichment strategies and mass spectrometry techniques have been made in the past few years, and studies comparing partial phosphoproteomes are emerging [5]. Even though both the incomprehensive nature of the data as well as differences in experimental procedures complicate comparative analysis, we can now start to exploit these data. Comparative analysis of phosphoproteomics data could increase our understanding of phosphorylation and the evolution of the phosphorylation network as a systems level property.

Not only do comparative analyses aid in elucidating the evolution of phosphorylation, but they also are a powerful tool with which to improve function prediction from sometimes noisy high-throughput datasets. For example, the use of conserved gene order has been shown to be a much stronger signal for protein function prediction than the order of genes in a single genome [6-8]. Similarly, the conservation of co-expression has been shown to aid function prediction from microarray data [9,10].

In this study we perform comparative analysis of phosphorylation events in eukaryotes. Our aim is to determine whether the quality of the data is sufficient to detect functionally significant overlap between high-throughput phosphoproteomics datasets, and to identify an evolutionarily significant pattern in this overlap. To address these questions, we compare recent high-throughput phosphoproteomics datasets of human, mouse, zebra fish, fruit fly, yeast, and plant. We determine the overlap between these datasets and show that this overlap is statistically, functionally, and evolutionarily relevant.

## Results

### Measuring the overlap in phosphoproteomes

We analyzed the overlap between high-throughput phosphoproteomics datasets from six species of eukaryotes. These datasets were created by different laboratories, using different experimental procedures (Table 1). In order to amend these datasets for comparative analysis, we imposed a relatively strict set of cutoffs on phosphopeptide calls in order to improve the uniformity and reduce noise caused by differences in scoring methods and thresholds (more details are provided in the Materials and methods section, below). The sizes of these individual datasets range from 724 to 3,296 (Table 1).

**Table 1:** Phosphoproteomics datasets

Species	Reference	Proteins (*n*)^a^	Sites (*n*)^a^
Human	[34]	1,419	3,296
Mouse	[23]	1,605	3,142
Fly	[14]	991	2,080
Yeast	[35]	481	850
Plant	[22]	470	724
Zebrafish	[11]	668	759

^a^Can be less than the number mentioned in the original papers, because we imposed a relatively strict set of cutoffs on phosphopeptide calls to improve the uniformity and reduce noise.

**Figure 1:**
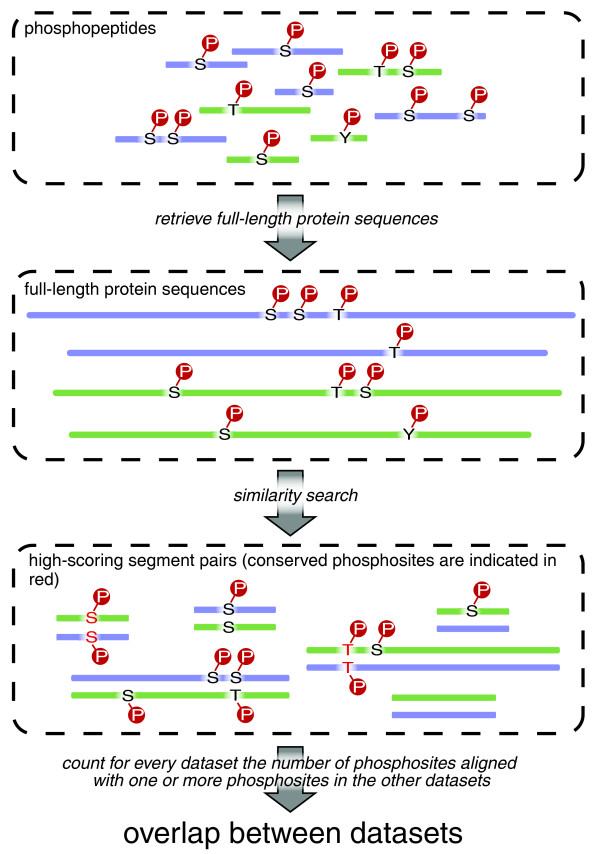
Workflow for determining conservation between two phosphoproteomics datasets. Black letters are amino acid residues, and a white p in a red circle indicates a phosphogroup. A more detailed description of this procedure can be found in the Materials and methods section.

We identified homologous sequences by an all-against-all Smith-Waterman search of all full-length proteins for which one or more phosphopeptides were present in the datasets. Phosphosites are considered homologous when a phosphosite in the query is aligned with the same type of phosphosite in the target sequence (workflow illustrated in Figure 1). For each dataset (the query) we counted the number of phosphorylation sites in the query datasets with at least one homolog in each of the target datasets (Table 2). The overlap between the datasets ranges from approximately 700 sites for human and mouse (two large datasets from closely related species) to a single site for fish and yeast (both distantly relate as well as two of the smallest datasets). Despite the virtually nonexistent overlap between fish and yeast, larger datasets of distantly related species exhibit considerable conservation; for example, mouse and plant share 27 phosphosites. We detect an overlap that is substantially larger than the overlap reported in specific phosphoproteomics experiments; the analysis conducted by Lemeer and coworkers [11] resulted in 50 phosphosites in zebrafish that had already been reported in human or mouse, whereas we find an overlap of more than 150.

**Table 2:** Number of query phosphorylation sites with at least one conserved site in the target species

Query\target	Plant	Fly	Human	Mouse	Yeast	Fish
Plant	×	9 (3.4)	13 (6.1)	27 (9.6)	3 (3.1)	4 (1.8)
Fly	9 (3.1)	×	85 (32.0)	72 (28.0)	4 (3.2)	35 (6.5)
Human	13 (5.6)	88 (33.7)	×	700 (155.5)	8 (6.3)	157 (27.6)
Mouse	27 (9.3)	79 (28.8)	706 (151.5)	×	13 (6.7)	151 (19.7)
Yeast	2 (2.8)	4 (3.1)	6 (5.9)	11 (6.4)	×	1 (1.6)
Fish	3 (1.5)	38 (6.5)	149 (26.0)	132 (18.9)	1 (1.6)	×

The number in parenthesis is average number of conserved sites of 1,000 randomization trials in which the position of phosphorylation sites were shuffled. Please note that the overlap is not symmetric, because a site in a query dataset can have multiple homologs in a target dataset.

### The overlap between phosphoproteomics sets is significant

In both a scenario in which the rate of evolution of reversible phosphorylation is so high that the species are too diverged to detect real homologous phosphosites, and when species completely re-wire their phosphoproteome after speciation, chance alone would result in a certain amount of overlap. We thus randomized for every protein in the datasets the positions of the phosphorylated residues across 1,000 trials and computed the average overlap. Note that this is a conservative null model, because it assumes that different species phosphorylate the same protein, whereas cases have been described in which different species use phosphorylation of different proteins for the regulation of the assembly of homologous protein complexes [3]. The observed overlap is larger than the average random overlap for almost all species comparisons (Table 2), strongly suggesting that the observed overlap is the result of significant evolutionary conservation.

Given the difficulty of formulating a null model for the significance of conservation between two species, we next considered the conservation of phosphorylation events over three or more species; if evolution plays no role in the overlap between datasets, then the chance of a specific site being conserved in one species will be independent of the presence or absence of that same site in other species. We thus compare the number of sites with homologs in two or more species with the number of sites that we would expect if we assume the chances of being conserved in different species to be independent (Table 3). For all datasets we observe that the number of sites observed in three, four, or five different species exceeds the number of sites expected assuming independence. Although we do not observe any phosphosites with homologs in all six species, we do observe a number of phosphorylation sites in *Arabidopsis thaliana *with homologs in one or more of the other datasets. These sites predate the evolutionary split between plants and ophistokonts, making them more than a billion years old [12].

**Table 3:** Number of sites found in three or more different species

	Three different species^a^		Four different species		Five different species	
	Observed	Expected^b^	Observed	Expected	Observed	Expected
Plant	9	1.55	2	0.02	2	0.00
Fly	33	7.55	13	0.18	2	0.00
Human	103	59.45	17	1.31	3	0.01
Mouse	106	63.59	23	1.80	4	0.02
Yeast	2	0.27	0	0.00	1	0.00
Fish	72	40.15	12	1.86	2	0.01

^a^Total number of species in which a phosphosite was present, including the query organism. We did not identify any sites with homologs in all six datasets. ^b^The number of expected sites assuming independent chances of conservation (the chance of a specific site being conserved in one species is independent of the presence or absence of that same site in other species).

### Relative overlap between phosphoproteomics data sets contains a strong evolutionary signal

Two independent tests suggest that the phosphorylation overlap is quantitatively significant. As a next step we tested for qualitative relevance by searching for a possible evolutionary pattern in the conservation of phosphoproteomics datasets. Specifically, we wondered whether a purported dynamic system level property such as the phosphorylation repertoire reflects the species phylogeny. However, interpreting the relative differences in overlap is far from trivial, because a myriad of both biological and technical factors, ranging from the sensitivity of the mass spectrometry analysis to experimental conditions under which phosphoproteomes were sampled, convolute a potential signal.

**Figure 2:**
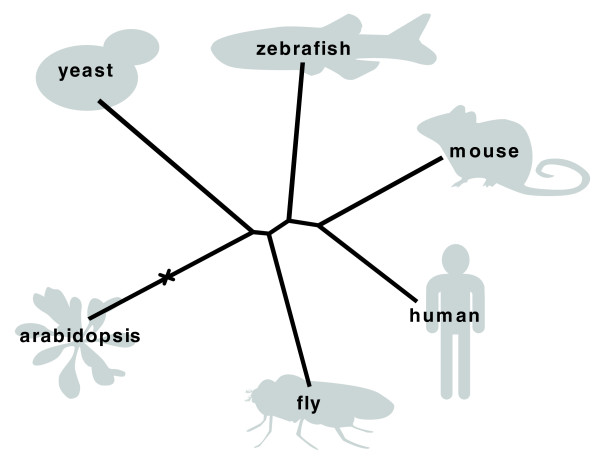
Phosphorylation follows phylogeny. The distance measure used in the construction of this neighbor-joining tree is (1 - relative overlap; described in detail in the main text). If the tree is rooted at the branch marked with the x, the topology of this tree is identical to the topology of the tree of life of these six species. The tree was generated with Quicktree [32] and visualized using Treeview [33].

In order to extract this potential signal, we determined the relative number of conserved phosphorylation sites by comparing the overlap with the number of sites that can potentially be conserved, given the proteins in the specific datasets: the relative overlap. This relative overlap can be obtained in a relatively straightforward manner by dividing the number of conserved phosphorylation events of the query and target datasets by the number of sites in the query dataset with one or more homologous positions in full-length proteins of the target dataset. We subsequently clustered the six species on the basis of their relative by the neighbor joining algorithm using 1 - (relative overlap) as the distance measure (Figure 2 and Additional data file 1). The topology of the unrooted tree that is the result of the neighbor-joining is identical to the topology of the tree of life for this small sample of six species.

**Figure 3:**
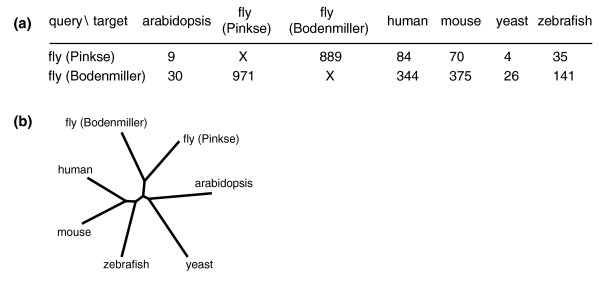
An additional dataset from fly. **(a) **Overlap between the additional fly dataset [13] and the original six datasets. **(b) **Neighbor-joining tree of the relative overlap between these seven datasets.

Variations in experimental conditions and protocols potentially obscure the evolutionary signal in the overlap between datasets. If this evolutionary signal is relatively strong, then the relative overlap between datasets from a single species should be greater than the relative overlap between datasets from different species. We determined the relative overlap between an additional dataset from fly [13] and the other six datasets (Figure 3a). This additional dataset contains many more phosphosites than the fly dataset that is already part of our analysis (the additional dataset contains 10,293 sites, as compared with the 2,080 of the original dataset), and the two datasets were constructed by different laboratories using different techniques [13,14]. Nevertheless, the relative overlap between both fly datasets is more than twice that with any of the other datasets (Figure 3a), and an extended neighbor-joining tree groups these two datasets together (Figure 3b). The relative overlap between the datasets is thus not only higher than expected by random chance; the relative overlap also follows phylogeny and thus contains a qualitatively strong and relevant evolutionary signal.

### Low-throughput experiments as a golden standard and conserved phosphosites and protein function

Conservation in sequence and gene order generally has functional meaning [8]. Low-throughput experiments are in general considered to be more reliable than high-throughput experiments, because they tend to be more suited to controls and validation. Several databases collect experimental data on reversible phosphorylation, for example Phospho.ELM [15] and Phosida [16]. Of all of the phosphosites in the human dataset, 2.5% have also been observed by a low-throughput experiment in the Phospho.ELM database; for the mouse dataset this is 2.0%. In contrast, 4.8% of the conserved sites in human and 4.2% of the conserved sites in mouse have been measured using low-throughput techniques, a significant increase (χ^2 ^test *P *< 0.0001). This observation shows that putative phosphorylation events with homologs in other high-throughput experiments are less likely to be false positives. This increase in reliability suggests that the overlap between phosphoproteomics datasets could be used as a tool with which to assess the reliability of putative phosphosites identified in high-throughput experiments, similar to the use of comparative methods for improving reliability of interactomes [17].

**Figure 4:**
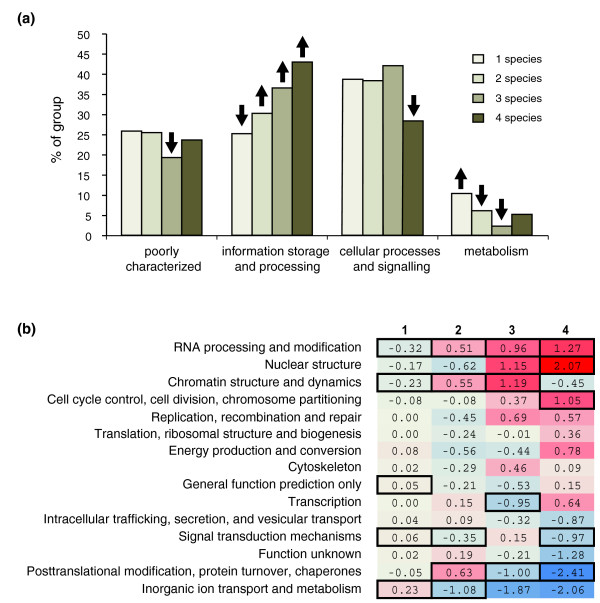
Functional classification of conserved phosphosites. **(a) **Main classes. The height of the bars represents the percentage of phosphosites with homologs in a specific number of different species (indicated by the color of the bar) belonging to the different classes. The black arrows indicate groups with homologs in a specific number of species that are significantly over-represented (arrows pointing up) or under-represented (arrows pointing down) compared with all phosphorylation events in that functional category. Significance was determined using a Fisher's exact test; scores with a *P *value below 0.05 after Bonferroni correction were considered significant. **(b) **Subclasses. The numbers in the cells are the fold increase of the fraction of phosphosites in that subclass relative to the fraction in that subclass of phosphosites without homologs in other species (^2^log [sites in *n *species] - ^2^log [sites in 1 species]). Over-representation is presented in red, and under-representation in blue. Only classes with a total of 80 or more sites and with at least one site found in a total of four species are shown. The black boxes indicate significant under-representation or over-representation (Fisher's exact test, *P *< 0.05 after Bonferroni correction).

Because some functional classes of proteins have been shown to be more conserved than others, we wondered whether this also holds for phosphorylation events. We utilized the functional classification provided by the Clusters of Orthologous Groups database [18] to study over-representation of biological processes among proteins with well conserved phosphosites (Figure 4a,b). These data reveal a clear functional trend in conserved phosphorylation sites; compared with sites that are found in only a single species, a relatively high percentage of phosphosites with homologs in two or more species are found in proteins with functions related to information storage and processing. Most striking is the over-representation of proteins that are involved in replication, chromatin structure, and cell cycle related processes, classes that contain functions that could be considered to be most fundamental for the survival of the cell. The presence of highly conserved phosphorylation events in these functional categories suggests that the fine-tuning mechanisms provided by phosphorylation arose early in evolution. Although based on these data we cannot exclude the possibility that this over-representation is influenced by other factors (for example, proteins with functions related to information storage and processing being more likely to have homologs in all six species studied), a link between conservation of phosphorylation events and protein function is in accordance with other observation (for example, protein function and duplication rate [19]).

Phosphorylation events identified in a single high-thoughput experiments are known to cluster outside globular domains, as meausured by PFAM [20]. Of the events we analyzed, 15% are found inside a domain predicted using domain predictions from the PFAM database [21]. When we only consider conserved phosphorylation events, this shows a slight increase to 17%. The similar percentage shows that the low occurrence of phosphoryalation in known globular domains holds true for evolutionarily conserved events, and hence is not the result of the presence of spurious phosphorylations in unconfirmed high-throughput data.

## Discussion

Both the incomprehensive nature of high-throughput phosphoproteomics experiments as well as idiosyncrasies of the experimental pipelines used by different laboratories complicate the comparison of high-throughput phosphoproteomics datasets. In addition, the data we are comparing result from experiments designed with different biological questions in mind; the plant experiment, for example, focuses on the phosphorylation of membrane associated proteins from cells grown in culture [22], whereas the mouse experiment uses protein extract from homogenized liver tissue [23]. All of these differences will undoubtedly introduce dissimilarities in the observed phosphoproteomes that do not reflect the evolutionary changes in phosphorylation networks between the different species, making the overlap that we found a minimal estimate. Randomization trials, functional bias in highly conserved phosphorylation events, and the relative differences in overlap between the six high-throughput phosphoproteomics datasets all suggest the overlap between these datasets to be biologically relevant, and we successfully identified the evolutionary signal in this overlap. We find a number of phosphorylation events that are likely to predate the evolutionary split between plants and animals. These sites thus appear to be ancient in origin, which is perhaps surprising, given that phosphorylation is thought to be a subtle regulatory mechanism.

Our work suggests that our understanding of reversible phosphorylation can be increased by comparing the results of high-throughput phosphoproteomics analysis with those from large-scale *in vitro *phosphorylation assays (for example [24,25]) or computationally predicted phosphoproteomes. In the current setup (comparing different mass spectrometry based high-throughput phosphoproteomics datasets), experimental idiosyncrasies already loom large over any comparison; hence, we did not include such datasets in this study. However, because we have now shown that the overlap is biologically significant, this restraint can be relaxed; comparative analysis in fact enables the use of the ever-increasing amount of data on phosphorylation obtained by high-throughput mass spectrometry experiments that were not designed specifically for this particular purpose.

Previous studies have described the conservation across multiple species of amino acid residues that are known to be phosphorylated in a specific organism [26] and have studied the conservation of the phosphorylation events themselves on a small scale (for example [27]). PhosphoBlast [4] provides a powerful tool with which to compare (phosphorylated) peptides, illustrated by the authors by comparing human and mouse phosphopeptide datasets. These studies revealed a relatively high conservation of amino acid residues that are known to be phosphorylated in one or more phosphoproteomics experiments, and identified a substantial overlap between the phosphoproteomes of different species. We extend this observation to larger evolutionary distances and show that the overlap is statistically, functionally, and evolutionarily relevant. These insights can applied, for example, to discriminating between noise and real phosphorylation events in high-throughput mass spectrometry experiments (analogous to the use of conserved gene order in the evaluation of BLAST significance scores [28]).

## Conclusion

The presence of functionally and evolutionarily significant overlap between high-throughput phosphoproteomics experiments allows the use of comparative phosphoproteomics in the prediction and evaluation of phosphorylation networks, similar to the established use of comparative genomics and transcriptomics in the elucidation of protein functions and biological networks. We expect the rapidly growing amount of data from high-throughput mass spectrometry analysis to make comparative phosphoproteomics a powerful tool in predicting, evaluating, and understanding reversible phosphorylation.

## Materials and methods

### Datasets

Table 1 lists the datasets compared in this study. Because our comparison of high-throughput datasets is already complicated by many factors, ranging from the incomprehensive nature of the data to differences in experimental procedures, we made an effort to keep putative false-positive phosphorylation sites from further confounding the analysis. We used criteria for filtering the input data that in many cases are more stringent than the criteria used in the original publications. Each dataset was preprocessed by removing all phosphopeptides with ambiguous sites (phosphogroups that could not be attributed to a specific amino acid residue), by removing peptides that could not be retraced unambiguously to one specific protein, and by applying a strict threshold on the peptide identification scores. For the human, fly, Arabidopsis, and zebrafish datasets we used a Mascot peptide score threshold of 35; for the mouse dataset we used an Ascore threshold of 19; and from the yeast dataset we took only phosphorylation sites with e-values of 1 × e^-04 ^or lower. For the additional fly dataset we used an dCn threshold of 0.1 and a PeptideProphet threshold of 0.9. Data handling was done with *ad hoc *Python scripts.

### Overlap

Homologous phosphosites were identified by doing an all-against-all similarity search using the Paralign implementation of the Smith-Waterman algorithm [29] of all of the full-length proteins for which one or more phosphopeptides were present in the datasets, followed by the identification of high-scoring segment pairs with an e-value of 1 × e^-10 ^or lower in which both the query and the target had the same type of phosphosites at exactly the same position in the alignment (a phosphorylated serine residue should be aligned with a phosphorylated serine residue). Because this procedure does not include any (reciprocal) best hit criteria, all we conclude is that similar sites are homologous; the exact nature of this relationship (orthologous, paralogous) remains unclear. We used a strict e-value threshold of 1 × e^-10 ^for the identification of homologous sequences. The use of a more liberal threshold would increase the overlap (we are now probably missing some homologous phosphorylation events because we did not consider the surrounding sequence to be sufficiently conserved) but would also introduce more noise into an already noisy dataset. In addition, a strict cutoff means that we do not erroneously assume convergently evolved small linear motifs to be homologous (motifs involved in recognition of phosphosites by their kinases tend to be extremely short [30]).

### Expected overlap between datasets assuming independence

The probability that a phosphorylation event in a query dataset is conserved in a target dataset is given by Equation 1.

(1) P(q ∈ O_Q, T_) = N_Q, T_/N_Q_

Where Q is the query dataset, q is a phosphorylation event in Q, T is the target dataset, ∈ means 'element of', ∉ means 'not an element of', O_Q, T _is the overlap of Q and T (events from Q with a homologous event in T), N_Q, T _is the number of events in O_Q, T_, and N_Q _is the total number of events in Q.

The probability that q has homologs in x of the target datasets is the sum of all possible combinations of presence and absence in all of the target datasets, given x. As an example, we consider target datasets A, B, and C. The probability *P *that q has homologs in two out of these three datasets is given by Equation 2.

(2) P(q|x = 2) = P(q ∈ O_Q, A _⋂ q ∈ O_Q, B _⋂ q ∉ O_Q, C_)+ P(q ∈ O_Q, A _⋂ q ∉ O_Q, B _⋂ q ∈ O_Q, C_) + P(q ∉ O_Q, A _⋂ q ∈ O_Q, B _⋂ q ∈ O_Q, C_)

Where *P*(q|x = 2) is the probability that q has homologs in two target datasets, and ⋂ is the 'and' operator.

The expected number of phosphorylation events from a query dataset with homologs in x target datasets is now given by Equation 3.

(3) E (x = i) = *P*(q|x = i).N_Q_

In which E is the expected value, and i is a number lower than the total number of datasets.

### Relative overlap

Relative overlap was calculated by dividing the number of conserved phosphorylation events of the query and target datasets by the number of sites in the query dataset with one or more homologous positions in the target dataset. We identified homologous positions using the results of the all-against-all similarity search described above; a site has a homologous position in a target dataset when the site is part of one or more high-scoring segment pairs in that dataset, irrespective of the specific residue type the site is aligned with.

### Domains

We identified known domains in the full-length sequence of all proteins with one or more phosphorylation events. Domains were identified with HMMER [31], using models provided by version 23 of the PFAM database [21]. The location of phosphorylation events relative to these domains was determined using python scripts.

## Authors' contributions

BS, AH, and JB conceived the study. BS, AH, and BvB participated in its design and coordination, and contributed to manuscript preparation. JB performed the analysis and drafted the manuscript. All authors read and approved the final manuscript.

## Additional data files

The following additional data are available with the online version of this paper. Additional data file 1 provides the number of conserved phosphosites per query phosphosite with one more homologous sites in the target dataset.

## Supplementary Material

Additional data file 1 Provided is the number of conserved phosphosites per query phosphosite with one more homologous sites in the target dataset. Click here for file
