# Supplementary material for: Comparative phosphoproteomics reveals evolutionary and functional conservation of phosphorylation across eukaryotes
Source: Genome Biol. 2008 Oct 1;9(10):R144. doi: 10.1186/gb-2008-9-10-r144 (PMC2760871; doi:10.1186/gb-2008-9-10-r144)
Supplement: Additional data file 1 — Provided is the number of conserved phosphosites per query phosphosite with one more homologous sites in the target dataset. [file gb-2008-9-10-r144-S1.pdf]

Supplementary file 1: calculating the expected number of sites with homologs in a specific number of other datasets

The probability that a phosphorylation event in a query dataset is conserved in a target dataset is given by equation 1.

(1) $P(q \in O_{Q,T}) = N_{Q,T} / N_Q$

Where Q is the query dataset, q is a phosphorylation event in Q, T is the target dataset, ∈ means "element of", ∉ means "not an element of", $O_{Q,T}$ is overlap of Q and T (i.e. events from Q with a homologous event in T), $N_{Q,T}$ is the number of events in $O_{Q,T}$, and N_Q is the total number of events in Q.

The probability that q has homologs in x of the target datasets is the sum of all possible combinations of presence and absence in all of the target datasets, given x. As an example we consider target datasets A, B and C. The probability P that q has homologs in two out of these three datasets is given by equation 2.

(2) $P(q \mid x{=}2) = P(q \in O_{Q,A} \cap q \in O_{Q,B} \cap q \notin O_{Q,C}) + P(q \in O_{Q,A} \cap q \notin O_{Q,B} \cap q \in O_{Q,C}) + P(q \notin O_{Q,A} \cap q \in O_{Q,B} \cap q \in O_{Q,C})$

Where $P(q \mid x{=}2)$ is the probability that q has homologs in two target datasets, and ∩ is the 'and' operator.

The expected number of phosphorylation events from a query dataset with homologs in x target datasets is now given by equation 3.

(3) $E(x{=}i) = P(q \mid x{=}i).N_Q$

In which E is the expected value, and i is a number lower than the total number of datasets.
